# P-2110. Strongyloides screening for heart transplant candidates in an endemic region: a single-center experience

**DOI:** 10.1093/ofid/ofaf695.2274

**Published:** 2026-01-11

**Authors:** Donna Dennis, Jessi Clark, Armaghan-e-Rehman Mansoor

**Affiliations:** University of Kentucky Healthcare, Lexington, Kentucky; University of Kentucky Healthcare, Lexington, Kentucky; University of Kentucky, Lexington, KY

## Abstract

**Background:**

*Strongyloides stercoralis* causes chronic intestinal infection and can reactivate in solid-organ transplant (SOT) recipients, leading to severe or fatal outcomes. The American Society of Transplantation (AST) guidelines recommends universal screening strategy in an endemic region. In the United States, the Appalachian region is endemic for *Strongyloides*, however screening data in SOT recipients has not been reported. We present data from patients undergoing evaluation for heart transplantation at a healthcare system serving a large portion of Appalachia.

**Table 1:**
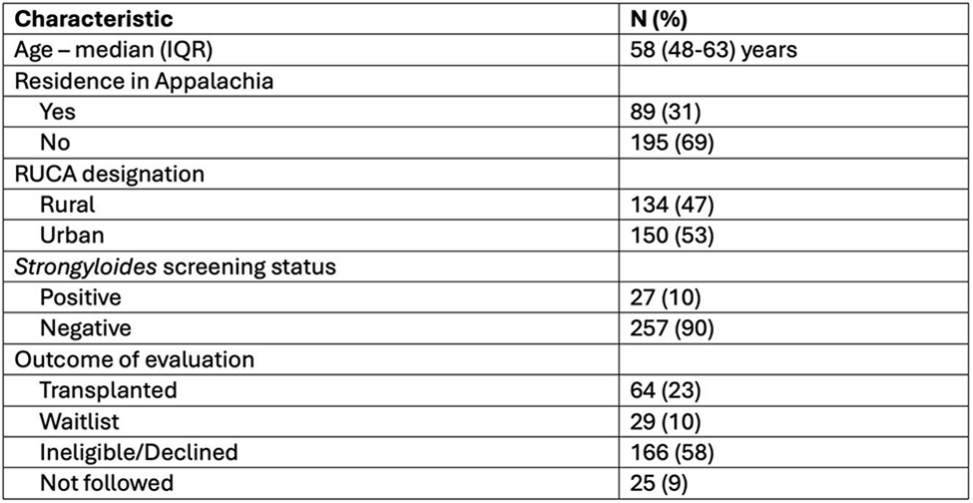
Demographics of prospective heart transplant recipients screened for Strongyloides

**Table 2:**
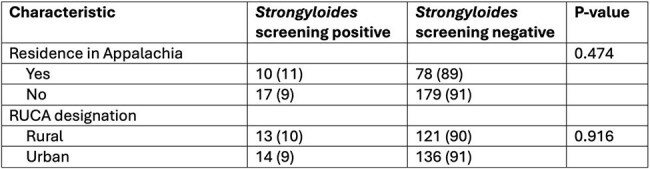
Association between residence and Strongyloides seropositivity

**Methods:**

Between January 2022 and December 2024, patients evaluated for heart transplantation were included. Screening for *Strongyloides* was performed using an ELISA immunoglobulin G (IgG) assay (ARUP; Salt Lake City, UT). Demographics, region of residence, and designation as rural vs urban region was recorded. Medical charts were reviewed for transplant outcomes.

**Results:**

A total of 311 transplant evaluations were performed in 293 patients, and serology was available for 284 patients. Residence within Appalachia was reported by 88 (31.0%) patients. Overall, 134 (47.2%) patients resided in a rural area, of whom 65 (22.9%) lived in Appalachia (Table 1). Serologic evidence of *Strongyloides* was found in 27 (9.5%) patients. Rural residents in Appalachia had the highest proportion of positive serology (14.2%), however there was no statistically significant difference compared to the overall cohort (14.2% vs 8.1%, p=0.143), Table 2. There was no significant association between positive serology and rural (9.7% vs 10.0%, p=0.934), or Appalachian residence (11.4% vs 8.7%, p=0.474). Six patients with positive serology were eventually transplanted, and all received therapy with ivermectin. There were no episodes of Strongyloidasis.

**Conclusion:**

Universal screening in an healthcare system serving a region within Appalachia showed 9.5% *Strongyloides* seroprevalence in heart transplant candidates. The strategy resulted in timely therapy for all transplanted individuals with exposure, and there were no reported instances of Strongyloidasis. While Appalachia is thought to be endemic for *Strongyloides*, our data raises concern for potential expansion of endemicity, which could be explored with future epidemiologic studies.

**Disclosures:**

All Authors: No reported disclosures

